# Meta-analysis on the safety and efficacy of long-term garlic consumption as an adjunctive treatment for hypertension

**DOI:** 10.3389/fnut.2025.1656809

**Published:** 2025-11-28

**Authors:** Yiyue Tang, Yunzhen Lei, Ming Xu, Qingxian Tu, Xiaochun Mao, Nanqu Huang, Qianfeng Jiang

**Affiliations:** 1 Department of Cardiovascular Medicine, The Third Affiliated Hospital of Zunyi Medical University (The First People’s Hospital of Zunyi), Zunyi, Guizhou, China; 2 Guizhou Aerospace Hospital, Affiliated Aerospace Hospital of Zunyi Medical University, Zunyi, Guizhou, China; 3 National Drug Clinical Trial Institution, The Third Affiliated Hospital of Zunyi Medical University (The First People’s Hospital of Zunyi), Zunyi, Guizhou, China

**Keywords:** garlic, hypertension, meta-analysis, blood pressure, cardiovascular disease

## Abstract

**Objectives:**

This systematic review and meta-analysis aimed to synthesize existing evidence, quantify the overall effect of garlic intervention on blood pressure, and explore potential variations in its effects under different population, formulation, and intervention duration conditions. This provides scientific evidence to support the clinical application of garlic in non-pharmacological interventions for hypertension.

**Methods:**

This systematic review and meta-analysis strictly adhered to the protocol registered with PROSPERO (CRD420251055848) and followed the guidelines outlined in the PRISMA statement. We conducted a comprehensive search of PubMed, Web of Science, Embase, and the Cochrane Library from their inception to May 10, 2025. Inclusion criteria were based on the PICOS framework: patients with a clear diagnosis of hypertension, treated with garlic or garlic products, compared with conventional medications or a placebo, with clearly defined outcome measures, and randomized controlled trials (RCTs). Meta-analysis was performed using Stata 14.0 software, assessing heterogeneity using the *Q*-test and I^2^ statistic, and publication bias using Egger’s test.

**Results:**

A total of 1,877 articles were retrieved, with 10 RCTs ultimately included in the meta-analysis. The results indicated a significant reduction in systolic blood pressure (SBP) (effect size: −4.21, 95% CI: −5.74 to −2.69, *P* < 0.001) and diastolic blood pressure (DBP) (effect size: −3.13, 95% CI: −4.42 to −1.84, *P* < 0.001) in the garlic intervention group compared to the control group. High-density lipoprotein (HDL) significantly increased (effect size: 0.31, 95% CI: 0.03 to 0.59, *P* = 0.03), and tumor necrosis factor-α (TNF-α) significantly decreased (effect size: −0.38, 95% CI: −0.72 to −0.04, *P* = 0.03). Subgroup analyses for SBP indicated significant reductions with a garlic dosage of 2.4 mg, an 8-weeks intervention, and in populations aged 50–60 years, with BMI of 18.5–24.9 and 30.0–34.9, baseline SBP of 130–139 mmHg and 140–149 mmHg, and baseline DBP of 70–79 mmHg and 90–100 mmHg. The incidence of adverse reactions in the garlic group was slightly higher, but not statistically significant, with gastrointestinal discomfort and bad breath being the most common side effects. Meta-regression analysis revealed that intervention duration, patient age, family history of cardiovascular disease, baseline SBP, and baseline DBP were positively correlated with the antihypertensive effect. S-allylcysteine (SAC) was negatively correlated, with an optimal dosage range of 0.5–1.5 mg.

**Conclusion:**

This meta-analysis suggests that garlic intervention has a significant effect on reducing blood pressure in certain populations, including those who are obese, aged 50–60, or have higher DBP. However, the overall impact on hypertension may be limited. Potential gastrointestinal and other adverse effects should be considered in clinical practice.

## 1 Introduction

Hypertension is a major risk factor for cardiovascular diseases worldwide. Chronic hypertension significantly increases the risk of stroke, myocardial infarction, heart failure, and chronic kidney disease (1). Although pharmacological treatment plays a central role in hypertension management, issues such as poor medication adherence, frequent adverse effects, and inadequate response to antihypertensive drugs in some patients have led to growing interest in non-pharmacological interventions (2). In recent years, garlic (*Allium sativum*), as a representative of traditional herbal medicine, has attracted widespread attention for its potential antihypertensive effects. Experimental studies have found that active compounds in garlic, such as S-allyl cysteine (SAC) and its derivatives, may exert antihypertensive effects through mechanisms such as improving endothelial function, promoting nitric oxide release, and inhibiting the activity of angiotensin-converting enzyme (ACE) (3, 4). Although several randomized controlled trials (RCTs) have evaluated the effects of garlic on hypertension, However, substantial heterogeneity exists in the current body of research, which may be attributed to imprecise participant selection, variations in intervention duration and methods, as well as inconsistencies in baseline characteristics of study populations. Such heterogeneity reduces the reliability and clinical applicability of the findings, thereby limiting their value for evidence-based practice (5).

Therefore, this study aims to provide an updated and more refined assessment of the antihypertensive effects of garlic by focusing specifically on hypertensive patients, thereby minimizing confounding effects from mixed populations. In addition, we conducted subgroup analyses based on multiple dimensions, including age, body mass index (BMI), gender, concurrent antihypertensive medication use, and comorbidities. To further explore potential sources of heterogeneity, we also performed meta-regression to identify factors that may influence the efficacy of garlic intervention in managing hypertension. By addressing these methodological improvements, our meta-analysis seeks to fill the existing gap in the literature and provide clearer, clinically relevant evidence for the non-pharmacological management of hypertension.

## 2 Materials and methods

### 2.1 Protocol

This systematic review and meta-analysis strictly adhered to the protocol registered with PROSPERO (CRD420251055848) and followed the guidelines outlined in the PRISMA statement.

### 2.2 Search criteria

#### 2.2.1 Inclusion criteria

The selection criteria in this meta-analysis were generated based on the PICOS principle as follows. Population: (1) Patients with a clear diagnosis of hypertension; (2) Treatment with garlic or garlic products; (3) Treatment with conventional medications or placebo; (4) Studies with clearly defined outcome measures; (5) Randomized controlled trials.

#### 2.2.2 Exclusion criteria

(1) Duplicate publications, (2) Animal testing, dissertations, basic research, reviews and case reports, and (3) Literature that cannot be read in full. (4) Articles that do not clearly give data on outcome indicators.

### 2.3 Search databases and strategy

PubMed, Scopus, Web of Science, Embase, and Cochrane Library were searched from their establishment to May 10, 2025. The search strategy is shown in Supplementary file.

### 2.4 Data extraction, and quality assessments

Literature screening and data extraction were independently performed by two researchers based on predefined inclusion and exclusion criteria. The process consisted of two stages: first, titles and abstracts were reviewed to exclude clearly irrelevant studies; second, full texts were assessed for eligibility. Discrepancies were resolved through discussion. The methodological quality of the included studies was evaluated using the Cochrane Risk of Bias 2.0 (ROB 2.0) tool, which assesses five domains: (1) randomization bias, (2) bias from deviations in interventions, (3) bias from missing data, (4) outcome measurement bias, and (5) bias in reporting results.

### 2.5 Statistical analysis

Meta-analysis was performed using Stata 14.0 software. Heterogeneity was assessed using the *Q*-test and the I^2^ test. I^2^ < 50% was considered low heterogeneity, and I^2^ ≥ 50% was considered high heterogeneity. If heterogeneity among the results of individual studies was low, a fixed-effects model was prioritized; otherwise, a random-effects model was used. Egger’s test was conducted to assess publication bias, and if publication bias was detected, the results were further examined using the clipped-patch method. Sensitivity analysis was employed to evaluate the robustness of the primary outcome indicators. Meta-regression was used to explore factors influencing the outcome indicators. A significance level of α = 0.05 was applied, and *P* < 0.05 was considered statistically significant.

## 3 Result

### 3.1 Literature search results

**FIGURE 1:**
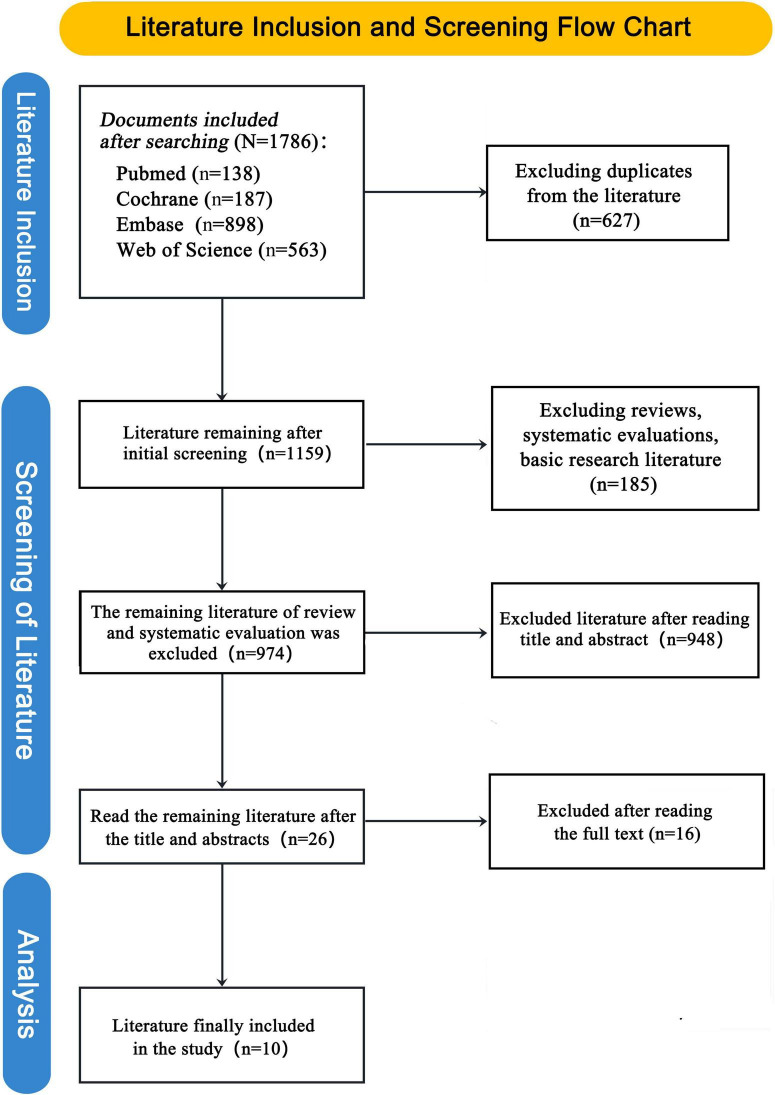
Screening flowchart.

The literature screening process began with 1,786 documents from four databases. After excluding duplicates (627) and irrelevant studies (185), 974 documents remained. Further screening reduced this to 26 studies, and after reading full texts and excluding 16 more, 10 studies were finally included in the analysis. See Figure 1 for details.

### 3.2 Description of included trials

A total of 10 studies were analyzed in this meta-analysis, with sample sizes varying across different groups. Participants’ ages ranged from 52 to 71 years, with mean ages typically falling between 54 and 66 years. The male ratio across the studies varied significantly, ranging from 16.7% to 100%. BMI values ranged from 23.0 to 31.0, with the majority of studies reporting a mean BMI between 25 and 30. Baseline SBP values varied across studies, with most reporting average SBP values ranging from 142 to 156 mmHg. Similarly, baseline DBP values ranged from 79.3 to 97.5 mmHg. The interventions included a variety of garlic-based treatments, such as daily garlic powder or extract supplements, as well as garlic-enriched chocolates and placebo treatments. Intervention durations typically ranged from 8 to 12 weeks, with some studies incorporating multiple intervention cycles. For further details, please refer to the Supplementary file.

### 3.3 Risk of bias assessments

**FIGURE 2:**
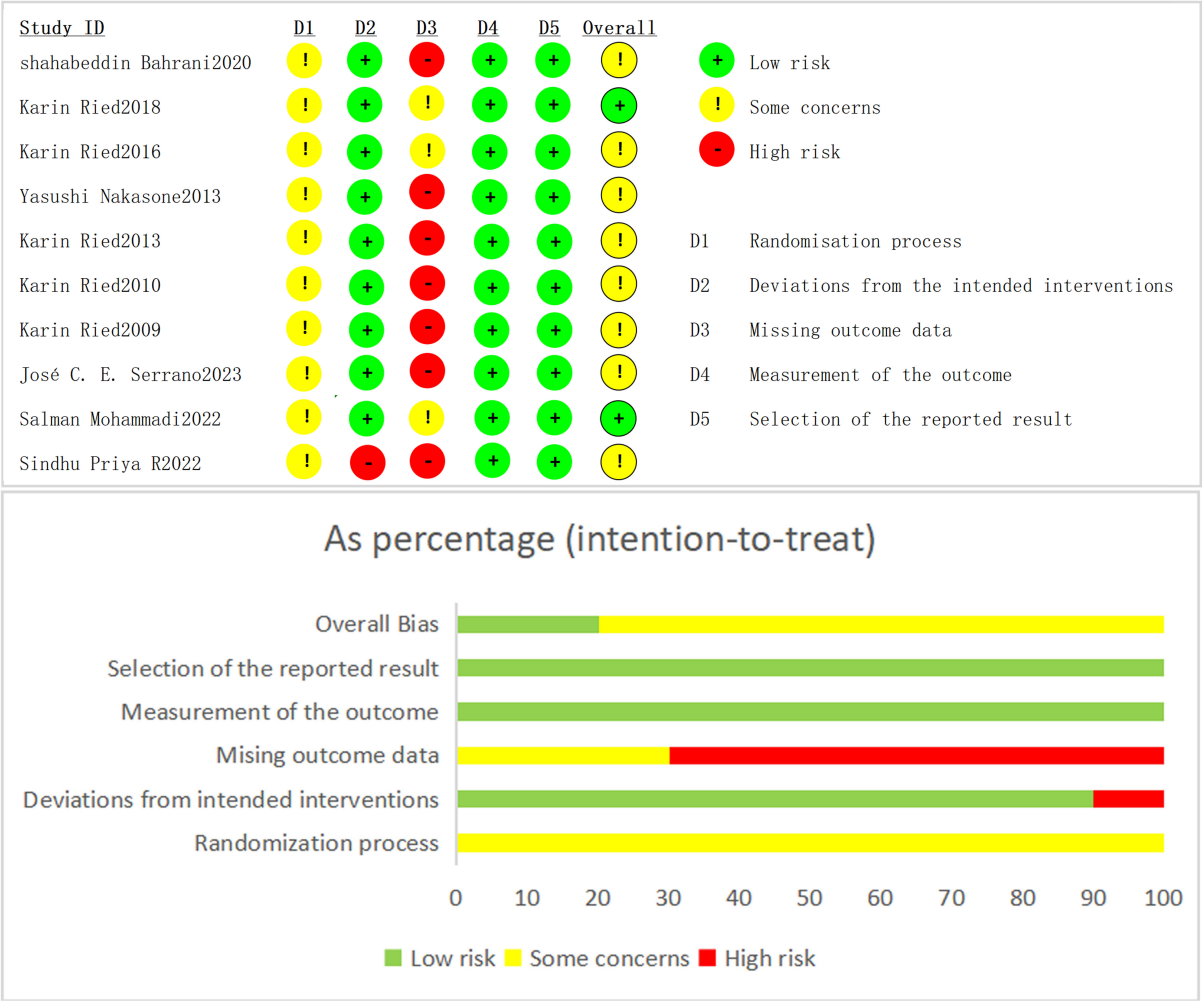
Literature quality assessment chart.

The risk of bias assessment revealed varying levels of quality across the studies in different domains. For overall bias, around 60% of the studies were deemed to have low risk, with 30% showing some concerns and 10% classified as high risk. In terms of selection of the reported result (D5), most studies (80%) were considered low risk, while 10% raised some concerns and another 10% had high risk. Measurement of the outcome (D4) was also generally strong, with 70% of studies at low risk, 20% showing some concerns, and 10% considered high risk. Regarding missing outcome data (D3), 40% of studies were low risk, 40% raised concerns, and 20% had high risk. Deviations from intended interventions (D2) showed that half of the studies had low risk, while 30% raised some concerns and 20% had high risk. Lastly, the randomization process (D1) was well-managed in most studies, with 90% at low risk and 10% having some concerns, with no studies rated as high risk. Overall, the quality of this article is reliable, Please refer to Figure 2 for details.

### 3.4 The effect of garlic on blood pressure and cardiovascular risk factors

**FIGURE 3:**
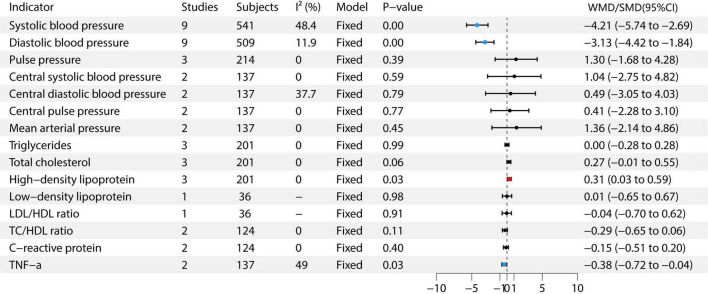
Forest plot of cardiovascular risk factors.

This meta-analysis included studies on various health indicators. For SBP, nine studies with 541 participants were included (6–14), with moderate heterogeneity (I^2^ = 48.4%), using a fixed-effects model. The effect size was −4.21 (95% CI: −5.74 to −2.69). For DBP, nine studies with 509 participants were included (6–14), with low heterogeneity (I^2^ = 11.9%), also using a fixed-effects model. The effect size was −3.13 (95% CI: −4.42 to −1.84). Pulse pressure was assessed in three studies with 214 participants, showing no heterogeneity (I^2^ = 0%) and using a fixed-effects model. The effect size was 1.30 (95% CI: −1.68 to 4.28) (6, 7, 12). For central SBP, two studies with 137 participants were included, showing no heterogeneity (I^2^ = 0%), and using a fixed-effects model. The effect size was −1.64 (95% CI: −2.75 to 4.82) (6, 7). Central DBP was assessed in two studies with 137 participants, with moderate heterogeneity (I^2^ = 37.7%), and a fixed-effects model was used. The effect size was 0.49 (95% CI: −3.05 to 4.03) (6, 7). Central pulse pressure and mean arterial pressure each included two studies with 137 participants, with no heterogeneity (I^2^ = 0%), and using a fixed-effects model. The effect sizes were 0.41 (95% CI: −2.28 to 3.10, *P* = 0.77) and 1.36 (95% CI: −2.14 to 4.86, *P* = 0.49) (6, 7), respectively. For triglycerides, three studies with 201 participants were included, showing no heterogeneity (I^2^ = 0%) and using a fixed-effects model. The effect size was 0.00 (95% CI: −0.28 to 0.28) (7, 9, 12). Total cholesterol was assessed in three studies with 201 participants, showing no heterogeneity (I^2^ = 0%) and using a fixed-effects model. The effect size was 0.31 (95% CI: 0.03 to 0.59) (7, 9, 12). HDL was included in three studies with 201 participants, also showing no heterogeneity (I^2^ = 0%) and using a fixed-effects model. The effect size was 0.31 (95% CI: 0.03 to 0.59) (7, 9, 12). Low-density lipoprotein (LDL) was assessed in one study with 36 participants, showing an effect size of 0.01 (95% CI: −0.65 to 0.67) (9). For C-reactive protein, two studies with 124 participants were included, with no heterogeneity (I^2^ = 0%) and using a fixed-effects model. The effect size was −0.15 (95% CI: −0.51 to 0.20) (7, 9). Finally, TNF-α was assessed in two studies with 137 participants, with moderate heterogeneity (I^2^ = 49%) and using a fixed-effects model. The effect size was −0.38 (95% CI: −0.72 to −0.04) (6, 7). The study found that garlic intervention significantly reduced SBP (WMD = −4.21, 95% CI: −5.74 to −2.69, *P* < 0.05), DBP (WMD = −3.13, 95% CI: −4.42 to −1.84, *P* < 0.05), total cholesterol (SMD = 0.31, 95% CI: 0.03 to 0.59, *P* = 0.03), HDL (SMD = 0.31, 95% CI: 0.03 to 0.59, *P* = 0.03), and TNF-α (SMD = −0.38, 95% CI: −0.72 to −0.04, *P* = 0.03). See Figure 3 for details.

### 3.5 The effect of garlic on SBP

**FIGURE 4:**
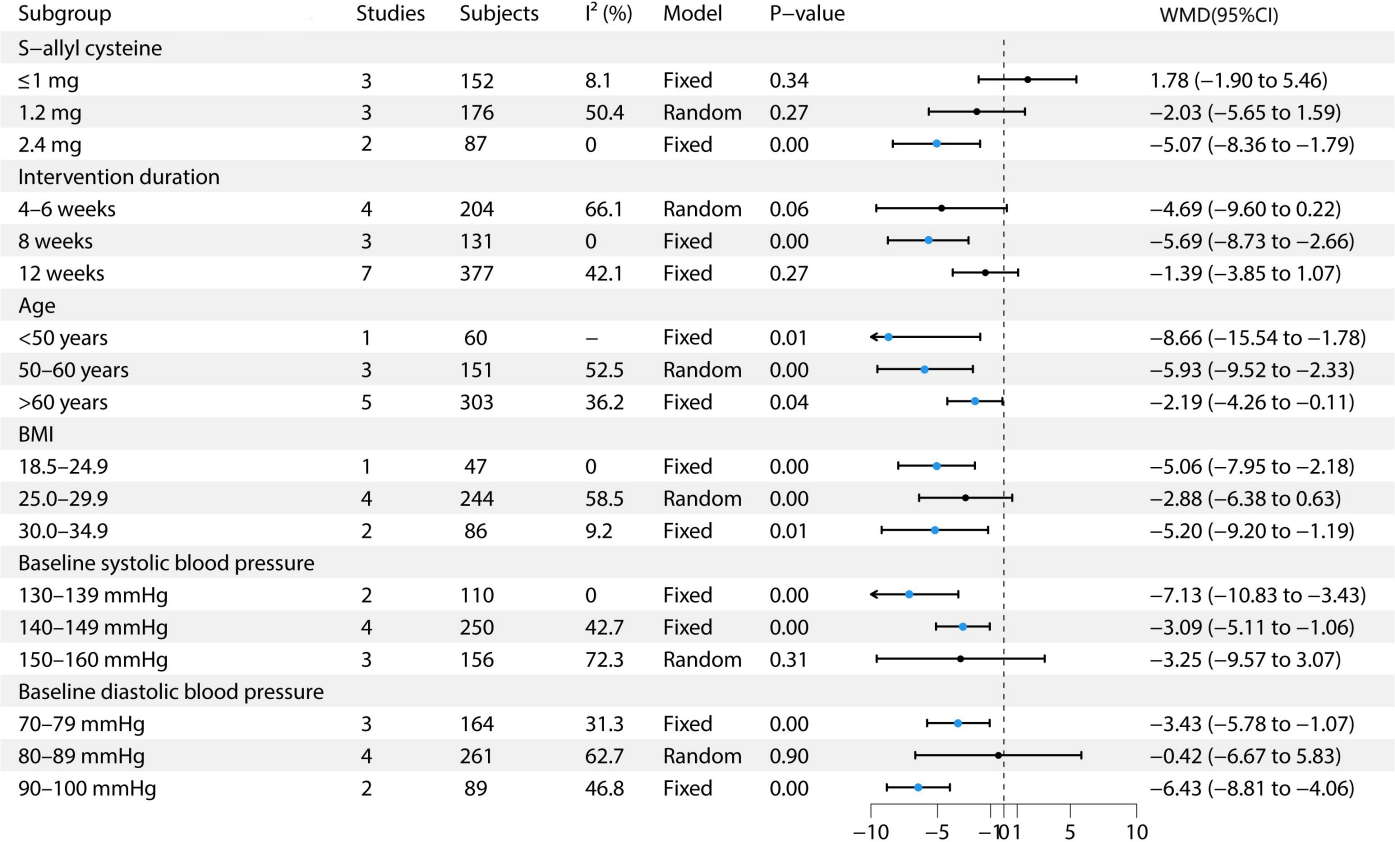
Forest plot of garlic on SBP.

This study conducted multiple subgroup analyses on the effect of garlic treatment on SBP. For S-allyl cysteine, the ≤1 mg dose included three studies with 152 participants, demonstrating low heterogeneity (I^2^ = 8.1%) and utilizing a fixed-effects model, with an effect size of 1.78 (95% CI: −1.90 to 5.46) (9, 12, 13). The 1.2 mg dose included three studies with 176 participants, exhibiting moderate heterogeneity (I^2^ = 50.4%) and using a random-effects model, resulting in an effect size of −2.03 (95% CI: −5.65 to 1.59) (6, 7, 13). The 2.4 mg dose included two studies with 87 participants, showing no heterogeneity (I^2^ = 0%) and utilizing a fixed-effects model, with an effect size of −5.07 (95% CI: −8.36 to −1.79) (13, 14). In terms of intervention duration, for the 4–6 weeks group, four studies with 204 participants were included, with moderate heterogeneity (I^2^ = 66.1%) and employing a random-effects model, resulting in an effect size of −4.69 (95% CI: −9.60 to 0.22) (8, 11, 13, 14). For the 8-weeks group, three studies with 131 participants showed no heterogeneity (I^2^ = 0%) and used a fixed-effects model, with an effect size of −5.69 (95% CI: −8.73 to −2.66) (11, 13, 14). For the 12-weeks group, seven studies with 377 participants showed moderate heterogeneity (I^2^ = 42.1%) and used a fixed-effects model, with an effect size of −1.39 (95% CI: −3.85 to 1.07) (6, 7, 9, 11–14). In the age subgroup analysis, the <50 years group included one study with 60 participants, utilizing a fixed-effects model, with an effect size of −8.66 (95% CI: −15.54 to −1.78) (10). The 50–60 years group comprised three studies with 151 participants, showing moderate heterogeneity (I^2^ = 52.5%) and using a random-effects model, with an effect size of −5.93 (95% CI: −9.52 to −2.33) (8, 9, 11). The >60 years group included five studies with 303 participants, with moderate heterogeneity (I^2^ = 36.2%) and used a random-effects model, showing an effect size of −2.19 (95% CI: −4.26 to −0.11) (6, 7, 12–14). In the BMI subgroup analysis, the BMI 18.5–24.9 group included one study with 47 participants, using a fixed-effects model, with an effect size of −5.06 (95% CI: −7.95 to −2.18) (11). The BMI 25.0–29.9 group included four studies with 244 participants, showing moderate heterogeneity (I^2^ = 58.5%) and using a random-effects model, with an effect size of −2.88 (95% CI: −6.38 to 0.63) (6–8, 13). The BMI 30.0–34.9 group included two studies with 86 participants, showing low heterogeneity (I^2^ = 9.2%) and utilizing a fixed-effects model, with an effect size of −5.20 (95% CI: −9.20 to −1.19) (9, 14). Regarding baseline SBP, the 130–139 mmHg group included two studies with 110 participants, using a fixed-effects model, with an effect size of −7.13 (95% CI: −10.83 to −3.43) (10, 14). The 140–149 mmHg group included four studies with 250 participants, showing moderate heterogeneity (I^2^ = 42.7%) and employing a random-effects model, with an effect size of −3.09 (95% CI: −5.11 to −1.06) (7, 11–13). The 150–160 mmHg group included three studies with 156 participants, exhibiting high heterogeneity (I^2^ = 72.3%) and using a random-effects model, with an effect size of −3.25 (95% CI: −5.97 to 3.07) (6, 8, 13). In the baseline DBP subgroup, the 70–79 mmHg group included three studies with 164 participants, with moderate heterogeneity (I^2^ = 31.3%) and utilizing a fixed-effects model, showing an effect size of −3.43 (95% CI: −5.78 to −1.07) (13, 14). The 80–89 mmHg group included four studies with 261 participants, exhibiting high heterogeneity (I^2^ = 62.7%) and using a random-effects model, with an effect size of −0.42 (95% CI: −6.67 to 5.83) (7, 9, 10, 12). The 90–100 mmHg group included two studies with 89 participants, showing moderate heterogeneity (I^2^ = 46.8%) and employing a fixed-effects model, with an effect size of −6.43 (95% CI: −8.81 to −4.06) (6, 8, 11). See Figure 4 for details.

### 3.6 The effect of garlic on DBP

**FIGURE 5:**
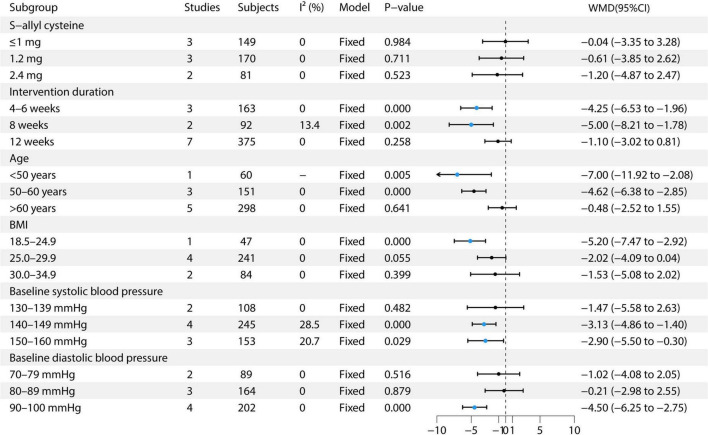
Forest plot of garlic on DBP.

This study performed multiple subgroup analyses to evaluate the effect of garlic treatment on DBP. For S-allyl cysteine, the 1 mg dose included three studies with 149 participants, resulting in an effect size of −0.04 (95% CI: −3.35 to 3.28) (9, 12, 13). The 1.2 mg dose included three studies with 170 participants, showing an effect size of −0.61 (95% CI: −3.85 to 2.62) (6, 7, 13). The 2.4 mg dose included two studies with 81 participants, with an effect size of −1.20 (95% CI: −4.87 to 2.47) (13, 14). Regarding intervention duration, the 4–6 weeks group included three studies with 163 participants, showing an effect size of −4.25 (95% CI: −6.53 to −1.96) (8, 11, 14). For the 8-weeks group, two studies with 92 participants showed an effect size of −5.00 (95% CI: −8.21 to −1.78) (11, 14). The 12-weeks group, which included seven studies with 375 participants, showed an effect size of −1.10 (95% CI: −3.02 to 0.81) (6, 7, 9, 11–14). In the age subgroup analysis, the <50 years group included one study with 60 participants, showing an effect size of −7.00 (95% CI: −11.92 to −2.08) (10). The 50–60 years group included three studies with 151 participants, showing an effect size of −4.62 (95% CI: −6.38 to −2.85) (8, 9, 11). The >60 years group included five studies with 298 participants, with an effect size of −0.48 (95% CI: −2.52 to 1.55) (6, 7, 12–14). In the BMI subgroup analysis, the BMI 18.5–24.9 group included one study with 47 participants, showing an effect size of −5.06 (95% CI: −7.95 to −2.18) (11). The BMI 25.0–29.9 group included four studies with 244 participants, showing an effect size of −2.88 (95% CI: −6.38 to 0.63) (6–8, 13). The BMI 30.0–34.9 group included two studies with 86 participants, showing an effect size of −5.20 (95% CI: −9.20 to −1.19) (9, 14). For baseline SBP, the 130–139 mmHg group included two studies with 108 participants, showing an effect size of −1.47 (95% CI: −5.58 to 2.63) (10, 14). The 140–149 mmHg group included four studies with 245 participants, showing an effect size of −3.13 (95% CI: −4.86 to −1.40) (7, 11–13). The 150–160 mmHg group included three studies with 153 participants, showing an effect size of −3.25 (95% CI: −5.97 to 3.07) (6, 8, 13). In the baseline DBP subgroup, the 70–79 mmHg group included two studies with 89 participants, showing an effect size of −1.02 (95% CI: −4.08 to 2.05) (13, 14). The 80–89 mmHg group included three studies with 164 participants, showing an effect size of −0.21 (95% CI: −2.98 to 2.55) (7, 9, 10, 12). The 90–100 mmHg group included four studies with 202 participants, showing an effect size of −4.50 (95% CI: −6.25 to −2.75) (6, 8, 11). The study found that the 2.4 mg S-allyl cysteine intervention for 8 weeks resulted in the most significant reduction in DBP. Notable reductions were observed in participants aged 50–60 years, with a BMI of 18.5–24.9 and 30.0–34.9, baseline SBP of 140–149 mmHg, and baseline DBP of 90–100 mmHg. See Figure 5 for details.

### 3.7 Safety evaluation

Findings from the meta-analysis reveal that the occurrence of negative side effects in the group receiving garlic extract was marginally greater compared to the control group (RR = 1.79, 95% CI: 1.18 to 2.72) (6, 11, 13–15). The primary side effects reported were mild digestive issues and halitosis.

### 3.8 Meta-regression analysis

**FIGURE 6:**
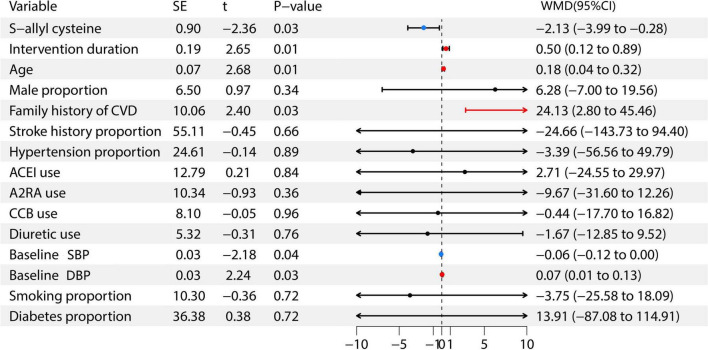
Meta-regression forest plot of factors affecting the antihypertensive effect.

Further analysis through meta-regression was conducted to explore the factors influencing the antihypertensive effect of garlic. In this meta-regression analysis, the included variables were: S-allyl cysteine, intervention duration, age, male proportion, family history of cardiovascular disease, stroke history proportion, previous hypertension proportion, ACEI use, A2RA use, CCB use, diuretic use, baseline SBP, baseline DBP, smoking proportion, and diabetes proportion (6–15). The weighted mean difference (WMD) represents the disparity between the average outcomes of the treatment group and the control group following the intervention. Findings indicated a positive relationship between S-allyl cysteine and baseline SBP with garlic’s antihypertensive effects, whereas factors such as intervention duration, age, family cardiovascular history, and baseline DBP showed a negative correlation. For further information, see Figure 6.

### 3.9 Sensitivity analysis

This study performed a sensitivity analysis on the results of systolic and DBP. The results showed that after excluding any single study, the pooled effect estimates and their 95% confidence intervals did not change significantly. This indicates that the results of this meta-analysis are robust and reliable, not overly influenced by any single study, thereby enhancing the credibility of the study’s conclusions. Specific results can be found in the Supplementary file.

### 3.10 Bias test

The publication bias of the studies was assessed using Begg’s and Egger’s tests. The analysis found no publication bias for both diastolic and SBP indicators. For further details, please refer to the Supplementary file.

## 4 Discussion

Hypertension, recognized as the foremost risk factor for cardiovascular diseases globally, has emerged as one of the most pressing public health challenges of the 21st century. Despite the widespread implementation of antihypertensive medications and lifestyle modifications, the rate of effective blood pressure management remains below 20%. This underscores ongoing challenges such as poor adherence to treatment, inappropriate medication choices, and a lack of personalized treatment strategies. Recent studies suggest that garlic may confer antihypertensive benefits through its active compounds, which enhance vascular function, reduce oxidative stress, and modulate critical signaling pathways.

The findings of this study revealed notable decreases in both SBP and diastolic blood pressure DBP within the garlic treatment group (SBP effect size: −4.21; DBP effect size: −3.13). Additionally, levels of HDL cholesterol rose significantly (effect size: 0.31), while TNF-α levels dropped (effect size: −0.38). Further analysis showed that garlic had marked antihypertensive effects at a dosage of 2.4 mg over an 8-weeks period in participants aged 50–60 years, with a BMI ranging from 18.5–24.9 and 30.0–34.9, as well as in those with mild hypertension. The primary side effects reported were mild gastrointestinal issues and halitosis.

In recent years, the antihypertensive effects of garlic as a natural remedy have been elucidated through numerous studies. Its bioactive compounds primarily exert antihypertensive effects by regulating vascular function, inhibiting oxidative stress, and intervening in key signaling pathways. The main bioactive compounds in garlic include SAC, allicin, AMS, and diallyl sulfide (DAS), with SAC being the predominant component (16). Organic sulfur compounds in garlic can enhance the activity of nitric oxide synthase (NOS) in endothelial cells. Through the NO/cGMP/PKG pathway, they reduce intracellular calcium concentrations, ultimately improving the state of vascular smooth muscle and lowering blood pressure (17). Meanwhile, allicin can act as a hydrogen sulfide (H_2_S) donor, inducing vascular smooth muscle cell hyperpolarization by activating K_ATP channels. This process reduces intracellular calcium concentrations and promotes vasodilation (18, 19). These vasodilatory mechanisms not only directly reduce blood pressure, but also indirectly alleviate physical damage to vascular endothelial cells by improving blood flow and reducing mechanical stress on the vessel walls, thereby providing a more stable environment for cellular DNA (20). At the same time, sulfur compounds in garlic, such as allicin, possess strong antioxidant activity. The primary mechanisms include inhibiting the RAS system, suppressing angiotensin II, and reducing the release of inflammatory factors (such as IL-6 and TNF-α) (21). Hypertension itself represents a state of sustained oxidative stress and chronic inflammation, characterized by the excessive production of reactive oxygen species (ROS). These ROS are primary culprits in oxidative DNA damage, as they attack the molecular structure of DNA, leading to base modifications (e.g., the formation of 8-hydroxy-2′-deoxyguanosine, 8-OHdG), strand breaks, and cross-linking. Such damage impairs cellular function and accelerates the aging process (22, 23). Through these processes, garlic can alleviate oxidative damage to the vascular endothelium, improve vascular function, and inhibit the formation of hypertension (24).

Additionally, studies have found that SAC can improve insulin sensitivity and glucose regulation. SAC improves renal sodium reabsorption by reducing insulin secretion and decreases excessive sympathetic nervous system activation by improving insulin resistance (25, 26). Besides direct influencing factors, garlic can also affect blood pressure by altering metabolic products in the blood. Matsutomo et al. compared the antihypertensive effects of AGE and its key components, S1PC and SAC. They found that both AGE and S1PC exhibited antihypertensive effects. Furthermore, S1PC significantly altered the plasma concentrations of metabolites such as betaine, tryptophan, and lysophosphatidylcholine, which are involved in blood pressure regulation (27).

**FIGURE 7:**
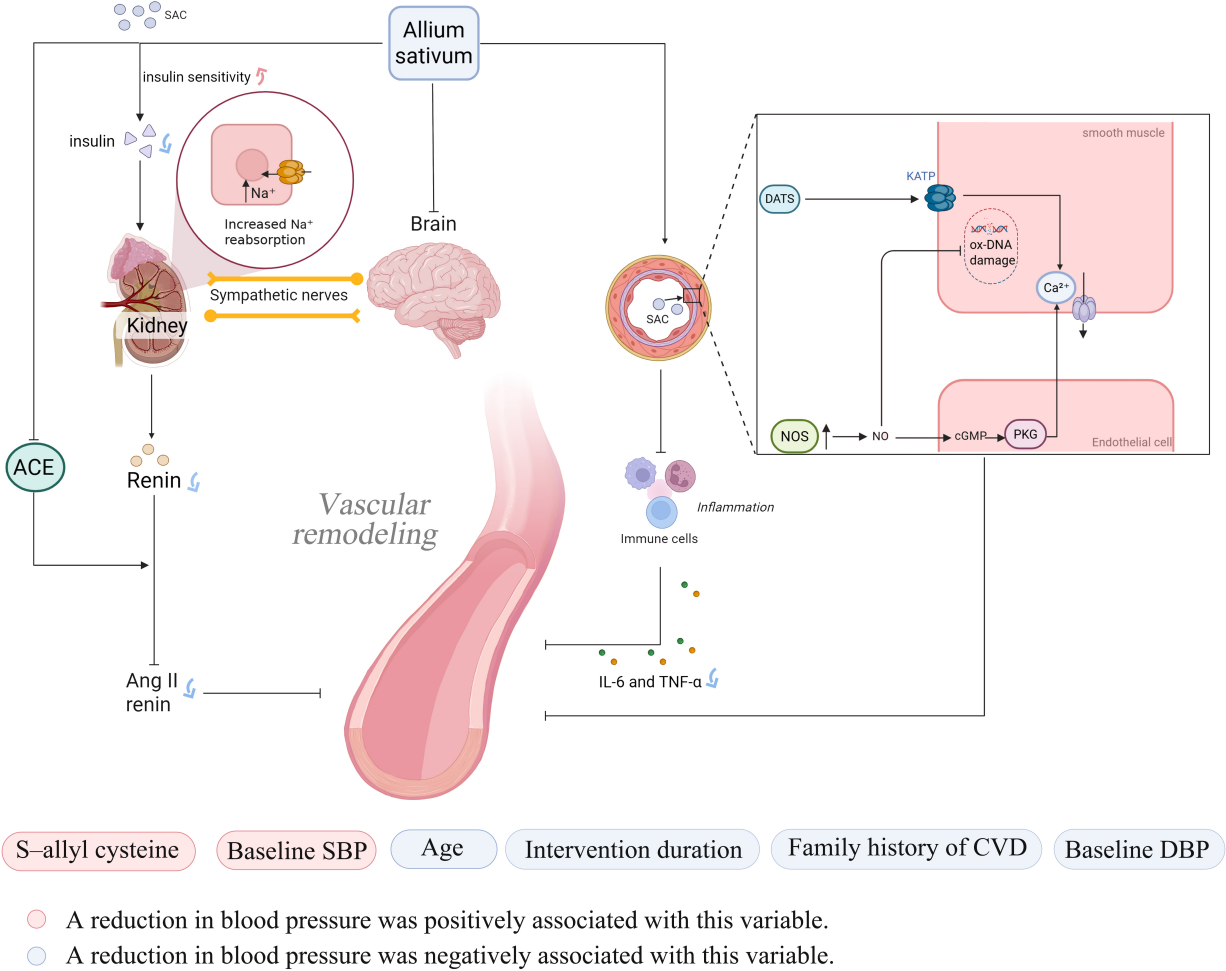
Mechanisms of garlic in the treatment of hypertension.

This study, in line with Saadh et al. found that garlic has an adjunctive antihypertensive effect (28), but further subgroup and meta-regression analyses revealed that the effect is short-term. This may be due to limited studies, individual variation, or the transient action and low bioavailability of allicin and SAC, which act through NO stimulation, RAS inhibition, and H_2_S-mediated vasodilation (29). The specific mechanism is illustrated in Figure 7.

In terms of safety, some studies have reported that high doses of garlic may cause allergic reactions, changes in platelet function and coagulation, as well as gastrointestinal discomfort (30), which may be attributed to the sulfur-containing compounds generated by high levels of allicin, which can bind to RAB18 protein and create a copper-enriched microenvironment, thereby inducing cuproptosis in hepatic stellate cells (31). Garlic oil supplementation (50 mg/kg) significantly prolonged bleeding time and thrombin time, and enhanced the activity of anticoagulant factors such as antithrombin III (32). However, there are differing views in the literature. One study found that in patients receiving warfarin treatment, the use of a higher concentration of aged garlic extract (10 mL/day, containing 14.7 mg of SAC) did not increase the incidence of bleeding compared to the placebo (33). Although this meta-analysis did not find that garlic use increases the risk of gastrointestinal bleeding in patients, adverse effects such as gastrointestinal discomfort were indeed observed.

In comparison with existing studies, which largely focus on patients with cardiovascular diseases, the composition of study populations may be a major source of heterogeneity observed in the results. Gadidala et al. found that garlic supplementation improved lipid metabolism and increased HDL levels in patients with coronary artery disease (34). These findings are consistent with the results of the present study, which further demonstrated that garlic intake can elevate HDL levels. This supports the potential role of garlic in ameliorating dyslipidemia. Similarly, a systematic review by Varade et al. focusing on individuals with metabolic syndrome also supports this perspective. Supporting this perspective, a systematic review by Varade et al. on individuals with metabolic syndrome demonstrated that garlic was particularly effective in improving lipid abnormalities and modulating inflammatory markers in this population (35). Kwak et al. further demonstrated the potential of garlic in improving glycemic control. However, the present study found that the presence or absence of diabetes did not significantly influence the blood pressure–lowering effects of garlic (36), which may be attributed to the shared pathophysiological mechanisms between hypertension and diabetes, such as chronic inflammation and oxidative stress. In studies focusing on patients with hypertension, this research extends the work of HAA Mizher et al.’s systematic review by further quantifying the antihypertensive effects of garlic through meta-analysis (37). Although Ma et al. in a meta-analysis investigating the effects of garlic on hypertensive patients, suggested that clinicians may consider recommending garlic consumption to lower blood pressure, the findings of the present study do not fully support this conclusion (38). The antihypertensive effects of garlic may vary depending on factors such as patient age, baseline blood pressure, presence of cardiovascular comorbidities, and duration of intervention. Therefore, whether garlic should be recommended as a therapeutic option for hypertension requires further stratified and quantitative investigation.

Our study further found that garlic extract was most effective in lowering blood pressure among individuals aged 50–60 years, whereas no significant effect was observed in those over 60. This may be because allicin and SAC promote immediate vascular dilation through the generation of NO and inhibition of ACE. Arterial stiffness naturally increases with age, and pulse wave velocity increases on average by 1.43 m/s per year over a period of 10 years (39). Although studies like those by Kyolic et al. have found that aged garlic extract can effectively restore arterial vitality and reduce pulse wave velocity, the persistent nature of arterial stiffness in elderly patients must be taken into account. Additionally, allicin (garlic’s active compound) is highly unstable in acidic environments, easily degrading into metabolites such as DAS. DAS can induce the proliferation and migration of arterial smooth muscle cells, which may contribute to lowering blood pressure (40), in elderly individuals, the reduction in gastric acid secretion and the decline in intestinal absorption function further decrease the bioavailability of active compounds. On the other hand, garlic extract has shown positive effects on overweight and obesity. In hyperlipidemic conditions, it can activate the sympathetic nervous system and inhibit eNOS through mechanisms involving the LOX-1 receptor, leading to hypertension. Previous studies have observed beneficial trends of garlic on inflammatory markers such as TNFα, total cholesterol, low-density lipoprotein cholesterol, and apolipoproteins (41, 42), which may be one of the reasons, but in this study, we only observed an impact of garlic on HDL in the population. This could be because the participants in this study were hypertensive patients rather than those with lipid metabolism disorders.

We also observed that garlic extract seemed to have a greater effect on DBP than on SBP. This could be related to the mechanisms of action of garlic. Allicin and SAC may activate potassium channels (K_ATP channels) by releasing H_2_S, leading to vascular smooth muscle hyperpolarization, lowering intracellular calcium ion concentration, and promoting vasodilation (19, 43). Garlic affects DBP by promoting vascular dilation and reducing peripheral vascular resistance. Studies have found that components in garlic help enhance the elasticity of blood vessels and reduce the impact of factors such as arteriosclerosis, potentially leading to a more pronounced reduction in DBP (44, 45). Overall, our study suggests that the impact of garlic intervention on hypertension may be limited. However, it could have more significant value for certain specific populations, such as those who are obese, aged 50–60, or have higher DBP. At the same time, the potential gastrointestinal and other adverse effects should not be overlooked.

This meta-analysis has several notable constraints: (1) Insufficient studies: The number of high-quality RCTs available is quite limited, which somewhat diminishes the robustness of the outcome measures. (2) Variability in subgroup evaluations: While the overall analysis indicates low variability, some subgroup evaluations reveal significant heterogeneity. There is a need for additional randomized studies to enhance the subgroup analyses. (3) Absence of long-term effect assessments: The studies primarily focus on intervention periods of 8–12 weeks, with a noticeable lack of research addressing long-term effects and follow-up, highlighting a gap in the existing RCT literature. (4) Inadequate information on garlic formulations: The constraints of the included studies prevent this meta-analysis from performing a thorough comparative evaluation of the efficacy and safety of various garlic formulations. The data available only permit an examination of S-allyl cysteine dosage, lacking a comprehensive analysis of the active ingredient concentrations, preparation methods, and bioavailability of other garlic products.

## 5 Conclusion

The findings of this meta-analysis indicate that the effectiveness of garlic treatment for hypertension might be restricted. Nevertheless, it may offer greater benefits for particular groups, including individuals who are overweight, those between the ages of 50–60, or those with elevated DBP. Additionally, it is important to consider the possible gastrointestinal issues and other negative side effects.

## Data Availability

The original contributions presented in this study are included in this article/Supplementary material, further inquiries can be directed to the corresponding authors.
