# Supplementary material for: Meta-analysis on the safety and efficacy of long-term garlic consumption as an adjunctive treatment for hypertension
Source: Front Nutr. 2025 Nov 28;12:1656809. doi: 10.3389/fnut.2025.1656809 (PMC12698422; doi:10.3389/fnut.2025.1656809)
Supplement: Supplementary file 1 [file Data_Sheet_1.docx]

**3.1 Literature search results**

| Database | #1 (Hypertension-related search) | #2 (Garlic-related search) | Combined Query (#1 and #2) |
| --- | --- | --- | --- |
| 138 (PubMed) | (Hypertension[MeSH Terms]) OR (Blood Pressure[MeSH Terms]) OR (Arterial Hypertension[MeSH Terms]) OR (High Blood Pressure[MeSH Terms]) | (Garlic[MeSH Terms]) OR (Allium sativum[MeSH Terms]) | #1 and #2 |
| 187 (Cochrane) | (Hypertension):ti,ab,kw OR (Blood Pressure):ti,ab,kw OR (Arterial Hypertension):ti,ab,kw OR (High Blood Pressure):ti,ab,kw | (Garlic):ti,ab,kw OR (Allium sativum):ti,ab,kw | #1 and #2 |
| 898 (Web of Science) | TS=(Hypertension) OR TS=(Blood Pressure) OR TS=(Arterial Hypertension) OR TS=(High Blood Pressure) | TS=(Garlic) OR TS=(Allium sativum) | #1 and #2 |
| 563 (Embase) | 'hypertension':ab,ti OR 'blood pressure':ab,ti OR 'arterial hypertension':ab,ti OR 'high blood pressure':ab,ti | 'garlic':ab,ti OR 'allium sativum':ab,ti | #1 and #2 |

**3.2 Description of included trials**

A total of 10 RCTs were included in this meta-analysis, involving participants across various age groups, including young adults, middle-aged individuals, and older adults. The participants’ body mass index (BMI) ranged from 23.0 to 31.0 kg/m², with sample sizes ranging from 16 to 163 individuals. Study populations included both male, female, and mixed-gender groups. The intervention types varied, including fresh garlic, aged garlic extract, dehydrated garlic paste, and encapsulated garlic extract. Control groups generally received placebo treatments, including placebo capsules or no active intervention. The duration of the interventions ranged from 6 weeks to 12 weeks. Further details are provided in Table 1.

| Author | Year | Group | Age  (Mean ± SD) | Male Ratio (%) | BMI  (Mean ± SD) | Baseline Systolic BP (Mean ± SD) | Baseline Diastolic BP (Mean ± SD) | Intervention Concentration | Intervention Duration |
| --- | --- | --- | --- | --- | --- | --- | --- | --- | --- |
| Shahabeddin Bahrani | 2020 | Garlic Group | 56.30 ± 7.55 | 42.40% | 28.05 ± 2.79 | 153.78 ± 9.43 | 97.27 ± 6.74 | 10g after breakfast and dinner daily | 6 weeks |
|  |  | Control Group | 54.31 ± 8.27 | 45.70% | 29.52 ± 4.13 | 148.71 ± 6.10 | 95.28 ± 4.99 |  |  |
| Karin Ried | 2018 | Garlic Group | 62.8 ± 9.3 | 44% | 28.6 ± 7.7 | 153.3 ± 16.4 | 93 ± 10.9 | 1.2g aged garlic extract powder and 1.2mg S-allylcysteine in 2 capsules daily | 12 weeks |
|  |  | Placebo Group | 61.9 ± 11.8 | 46% | 29.7 ± 6.2 | 144.3 ± 14.2 | 90 ± 12.3 | 2 placebo capsules daily |  |
| Karin Ried | 2016 | Garlic Group | 63.3 ± 9.9 | 56% | 27.3 ± 4.9 | 148.7 ± 15.3 | 89.9 ± 11.7 | 1.2g aged garlic extract powder and 1.2mg S-allylcysteine in 2 capsules daily | 12 weeks |
|  |  | Placebo Group | 61.5 ± 13.0 | 50% | 28.3 ± 4.9 | 142 ± 9.4 | 87.8 ± 9.1 | 2 placebo capsules daily |  |
| YASUSHI NAKASONE | 2013 | Garlic Group | 54 ± 8 | 56.50% | 23 ± 2 | 142 ± 6 | 91 ± 6 | 300mg dehydrated garlic paste daily | 12 weeks |
|  |  | Placebo Group | 53 ± 9 | 54.20% | 25 ± 3 | 142 ± 6 | 92 ± 6 | None |  |
| K Ried | 2013 | Garlic Group | 70.4 ± 13.1 | 45.90% | 28.8 ± 4.0 | 149.4 ± 3.0 | 75.9 ± 2.8 | 960mg aged garlic extract and 2.4mg S-allylcysteine in 4 capsules daily | 12 weeks |
|  |  | Placebo Group | 71.5 ± 10.9 | 45.90% | 29.9 ± 4.5 | 148.6 ± 3.0 | 76.0 ± 2.8 | Placebo capsules matching experimental capsules in size, color, and scent |  |
| K Ried | 2010 | Garlic Group | 66 ± 9 | 68% | 31 ± 5.8 | 146.2 ± 10.5 | 79.3 ± 11.8 | 960mg aged garlic extract and 2.4mg S-allylcysteine in 4 capsules daily | 12 weeks |
|  |  | Placebo Group | 66 ± 9 | 68% | 29.1 ± 4.7 | 151.1 ± 10.4 | 80.4 ± 7.9 | Placebo capsules matching experimental capsules in size, color, and scent |  |
| K Ried | 2009 | 900mg Kwai Group | 52.2 ± 2.5 | 100% | 25.3 ± 0.8 | 156.9 ± 2.8 | 97.5 ± 1.0 | 900mg garlic daily | 8 weeks |
|  |  | Placebo Group | 52.7 ± 2.5 | 100% | 26.0 ± 1.0 | 154.8 ± 3.2 | 96.1 ± 1.3 | No active ingredient |  |
| José C. E. Serrano | 2023 | Garlic Group | 63.7 ± 5.8 | 33.90% | NA | 148.8 ± 22.9 | 85.7 ± 11.2 | Optimized black garlic extract with 0.25mg SAC daily | 12 weeks |
|  |  | Placebo Group | 64.1 ± 5.9 | 34.50% | NA | 145.5 ± 17.2 | 85.3 ± 10.8 | Placebo tablet daily |  |
| Salman Mohammadi | 2022 | Garlic Group | 51.94 ± 9.6 | 56.30% | 30.5 ± 4.07 | 125.5 ± 18.7 | 89.2 ± 11.5 | 5g chocolate daily with 0.65g encapsulated fermented garlic extract | 12 weeks |
|  |  | Placebo Group | 51.9 ± 7.7 | 30% | 29.65 ± 3.96 | 124.47 ± 15.6 | 84.33 ± 9.4 | 5g placebo chocolate daily |  |
| Sindhu Priya R | 2022 | Garlic Group | 31-50 years (36.7%) | 16.70% | NA | 131.67 ± 12.62 | 86.33 ± 8.09 | NA | NA |
|  |  | Control Group | 19-30 and 31-50 years (30%) | 40% | NA | NA | NA | NA | NA |

Table 1: Study Characteristics

**3.9 Sensitivity Analysis**

A sensitivity analysis was conducted on systolic blood pressure using a leave-one-out method. The results showed that the range of effect estimates for systolic blood pressure varied from -8.11 to -3.18, indicating that most studies had a minimal impact on the overall effect estimate. The results demonstrate good robustness. See Figure 1 for details. A sensitivity analysis was conducted on diastolic blood pressure using a leave-one-out method. The results showed that the range of effect estimates for diastolic blood pressure varied from -4.78 to -1.00, indicating that most studies had a minimal impact on the overall effect estimate. The results demonstrate good robustness. See Figure 2 for details.


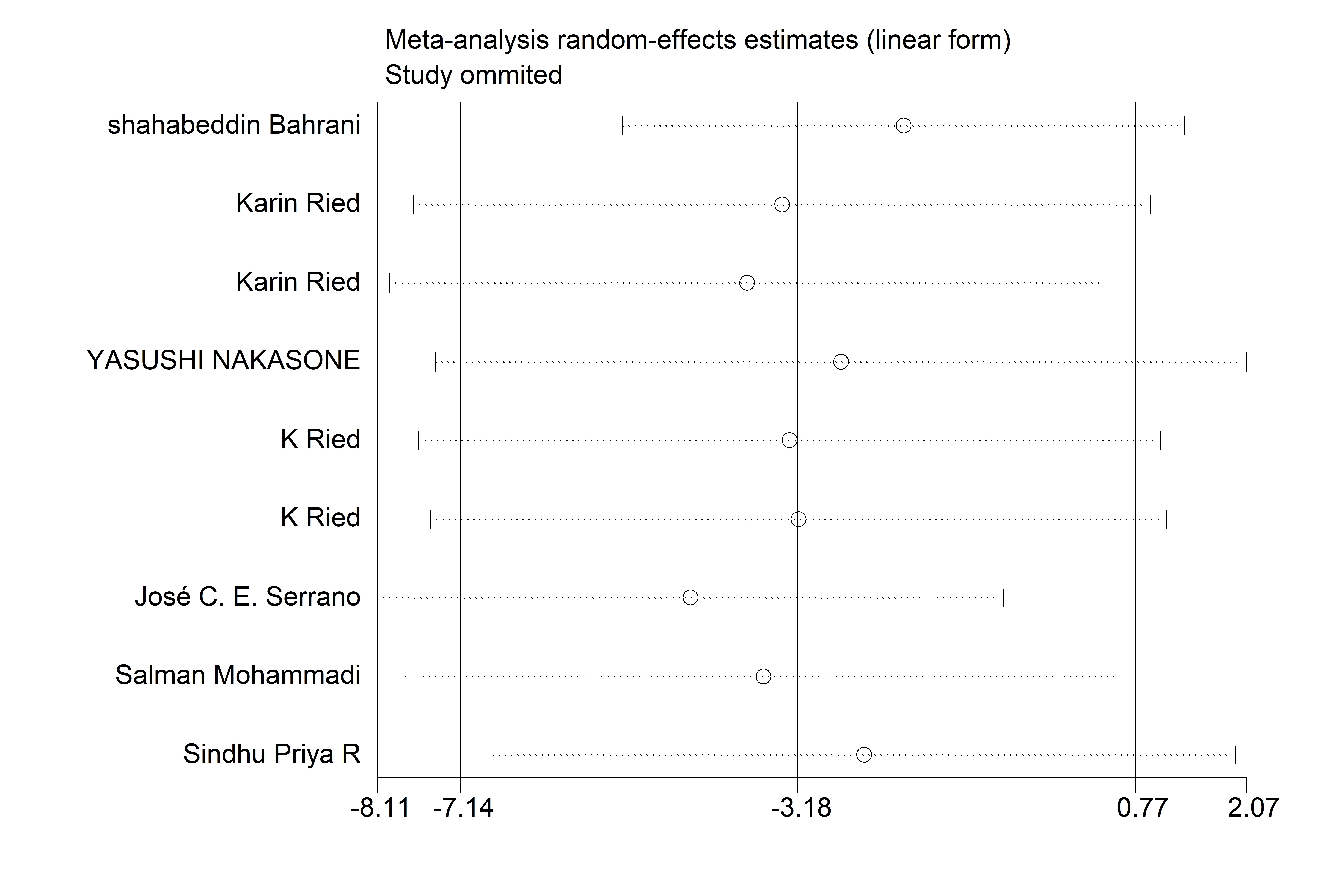


Figure 1: Sensitivity Analysis of Systolic Blood Pressure.

Figure 2: Sensitivity Analysis of Diastolic Blood Pressure.

**3.10 Bias Test**

The table presents the publication bias test results for systolic and diastolic blood pressure. For systolic blood pressure (mmHg), the z-value of Begg's test was 1.04, with a p-value of 0.297, indicating no significant publication bias according to Begg's test. The statistic for Egger's bias test was 3.69, with a p-value of 0.065. Although there was a potential trend for publication bias, it did not reach statistical significance at the 0.05 level. For diastolic blood pressure (mmHg), the z-value of Begg's test was 0, with a p-value of 1, indicating no evidence of publication bias. The statistic for Egger's bias test was 1.56, with a p-value of 0.248, also suggesting no significant publication bias for diastolic blood pressure.

| Group | n | Begg's z | Begg's p | Egger's Bias | Egger's p |
| --- | --- | --- | --- | --- | --- |
| SBP (mmHg) | 9 | 1.04 | 0.297 | 3.69 | 0.065 |
| DBP (mmHg) | 9 | 0 | 1 | 1.56 | 0.248 |

Table 2: Bias Test Table.
